# A Wearable Wireless Sensor Network for Indoor Smart Environment Monitoring in Safety Applications

**DOI:** 10.3390/s17020365

**Published:** 2017-02-14

**Authors:** Diego Antolín, Nicolás Medrano, Belén Calvo, Francisco Pérez

**Affiliations:** Grupo de Diseño Electrónico (I3A), Departamento de Ingeniería Electrónica y Comunicaciones, Universidad de Zaragoza, C/Pedro Cerbuna 12, Zaragoza 50009, Spain; nmedrano@unizar.es (N.M.); becalvo@unizar.es (B.C.); fperezpellicena@gmail.com (F.P.)

**Keywords:** wearable sensor node, wearable wireless sensor network (W-WSN), CO_2_ smart detection, safety applications, remote web application

## Abstract

This paper presents the implementation of a wearable wireless sensor network aimed at monitoring harmful gases in industrial environments. The proposed solution is based on a customized wearable sensor node using a low-power low-rate wireless personal area network (LR-WPAN) communications protocol, which as a first approach measures CO_2_ concentration, and employs different low power strategies for appropriate energy handling which is essential to achieving long battery life. These wearables nodes are connected to a deployed static network and a web-based application allows data storage, remote control and monitoring of the complete network. Therefore, a complete and versatile remote web application with a locally implemented decision-making system is accomplished, which allows early detection of hazardous situations for exposed workers.

## 1. Introduction

The growing advances in the last two decades in low-power wireless communications, the constant downsizing in electronic devices and the progressive increase of computational power in low-cost microcontrollers have fostered the emergence of cyber-physical systems (CPSs). A CPS [1,2] consists of a physical structure (a natural area, house, facility or even a human body) where a set of sensors monitors the main system parameters in order to obtain maximum knowledge and/or take advantage, with a minimum impact to the environment. Sensors interact with each other in order to obtain high-level information of the monitored physical system, to control some internal processes or to improve some characteristics, without the requirement of interaction with a central control system [3,4,5,6,7,8].

In safety monitoring, in industrial environments the usual approach lies in deploying static wireless sensor nodes in the area of interest. In this way, ambient monitoring systems based on wireless sensor networks (WSN) can be found in different scenarios such as refrigerated chambers [9], chemical production plants [10], or in modern steel mills to detect carbon monoxide [11]. In such potentially dangerous scenarios, continuous monitoring using wearable body sensor networks—added to work wear—will increase early detection of threatening situations for exposed workers. Wearable wireless sensor network (W-WSN) systems are a particular CPS case where sensors are deployed on the user clothing and/or body to monitor physiological parameters, environmental conditions, or both. Unlike conventional WSNs, W-WSNs consist of fewer and smaller nodes, covering less space. A typical wearable wireless sensor network consists of several wearable nodes connected between them or connected to a static WSN. Each wearable node includes low-power sensors, a wireless transceiver, electronic processing elements (microcontroller and interface systems) and the power supply unit, which must be miniaturized, lightweight and long lasting.

This paper presents a complete W-WSN system based on a custom wearable sensor node using a low-power low-rate wireless personal area network LR-WPAN communications protocol, intended for monitoring ambient parameters in potentially harsh environments where carbon dioxide (CO_2_) gas leaks may occur. The proposed node contains two main sensors: CO_2_ concentration is measured by an IRC-A1 gas sensor from Alphasense (Essex, UK) with a measurement range from 0 to 50,000 ppm [12]; temperature and humidity are complementary parameters, measured by a SHT11 digital humidity sensor (Sensirion AG, Zurich, Switzerland) [13]. These nodes are designed to be connected to a deployed static network. A web-based application allows data storage, remote control and monitoring of the complete network, therefore achieving a complete and versatile remote web application with a locally-implemented decision-making system.

The paper is structured as follows: Section 2 provides a brief background to better situate the present work. Section 3 presents the description of the sensor nodes, including the core hardware, microcontroller programing technologies and networking. Within the implementation strategies, special attention has been paid to reach optimal energy handling, essential to achieving long battery life. Section 4 comprises experimental results. Section 5 explains the operation of the complete remote web application system and finally, Section 6 presents the conclusions and future work.

## 2. Previous Work

The proposed wearable device is based on a preliminary work [14], which served as the starting point to implement this optimized device that includes several both hardware and software improvements: an integrated battery charge system, static voltage scaling (SVS) and dynamic frequency scaling (DFS) techniques, and software routines that allow automatic node connection to a previously-deployed WSN. The CO_2_ sensor board includes a custom low-power astable circuit that excites the gas sensor, so the sensor response time keeps constant and independent of the microcontroller power mode. In addition, an acoustic buzzer has been included to advise the user about hazardous CO_2_ levels in the nearby environment.

The wireless sensor network used as a fixed infrastructure where portable sensors can be added is based on a work by Antolín et al. [15]. When deployed indoors, the power consumption requirements of this static WSN can be relaxed by using suitable medium-sized batteries or even a mains power connection, so the duty cycle and number of physical external parameters to be measured per node can be increased without jeopardizing the system operating life. Static nodes are configured as router devices, while wearable sensors are configured as end devices. This guarantees that wearable nodes are always connected to the network through static devices, which allows its physical location to allow early detection of gas leakage.

## 3. Wearable Wireless Node Architecture

### 3.1. Wearable Sensor Node Hardware Design

**Figure 1:**
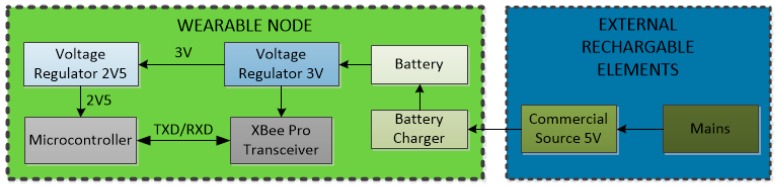
Wearable sensor node block diagram.

**Figure 2:**
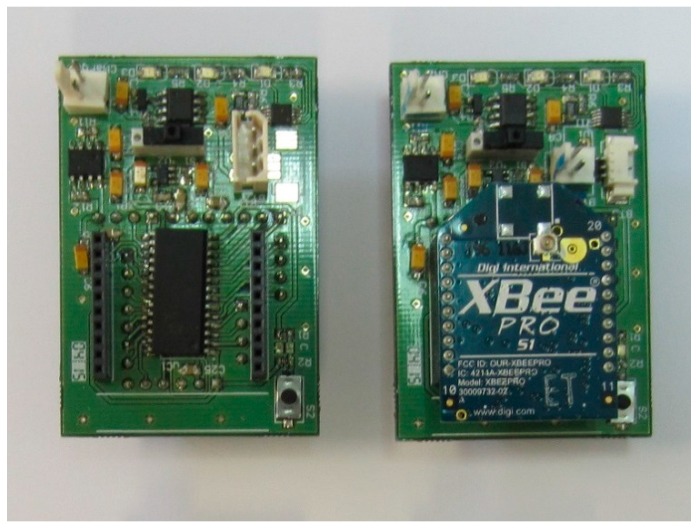
Wearable sensor node photograph: (**Left**) without XBee; (**Right**) with XBee.

To achieve a truly wearable sensor device, key hardware design requirements are minimum size, efficient power management and a compact rechargeable battery system. Figure 1 shows the block diagram of the developed sensor node, and Figure 2 shows its photograph. It consists of a PIC18F26J50 8-bit microcontroller (µC) (Microchip Technology Inc., Chandler, AZ, USA), with nanoWatt technology, selected for its cost-characteristics tradeoff and low power consumption. It owns several energy-saving working modes and independent clock lines that can be addressed to different peripherals according to its timing requirements. It can be dynamically powered at a voltage ranging from 2.0 V to 3.6 V, with a quiescent current of 6.2 µA in non-sleep modes.

The microcontroller manages the node operation, i.e., sensor acquisition; data collection and storage; building of the data frame to be sent by the radio frequency (RF) transceiver; sending of the data frame to the RF transceiver through an RS-232 protocol; and monitoring of the node state (energy mode, initialization, etc.). The node RF transceiver is an XBee module with a wire antenna to reduce the size. It includes the DM-24 firmware from DigiMesh that works in the 2.4 GHz Industrial-Scientific-Medical (ISM) band with an IEEE 802.15.4 protocol running in the transceiver, making compatible the connection of this device to a deployed WSN and allowing drawing up of its communications structure.

The sensor node includes a novel and compact power supply system similar to those available in smartphones and other portable consumer electronic devices. It consists of a MCP73833 battery charger circuit from Microchip, a rechargeable battery and two low dropout (LDO) regulators to provide the power voltage levels required by the different components. The selected battery is a 3.7 V and 800 mAh Lithium Polymer (LiPo) LP-573442-1S-3 that meets the W-WSN node energy requirements with a small size. The voltage regulators provide stable, well-defined voltage levels from the decreasing battery voltage: an MCP1725 LDO voltage regulator (Microchip Technology Inc., Chandler, AZ, USA) provides a 3 V constant voltage to power the XBee RF transceiver. The second LDO regulator (TC1015 also from Microchip) provides a constant 2.5 V level to power the rest of node electronics.

**Figure 3:**
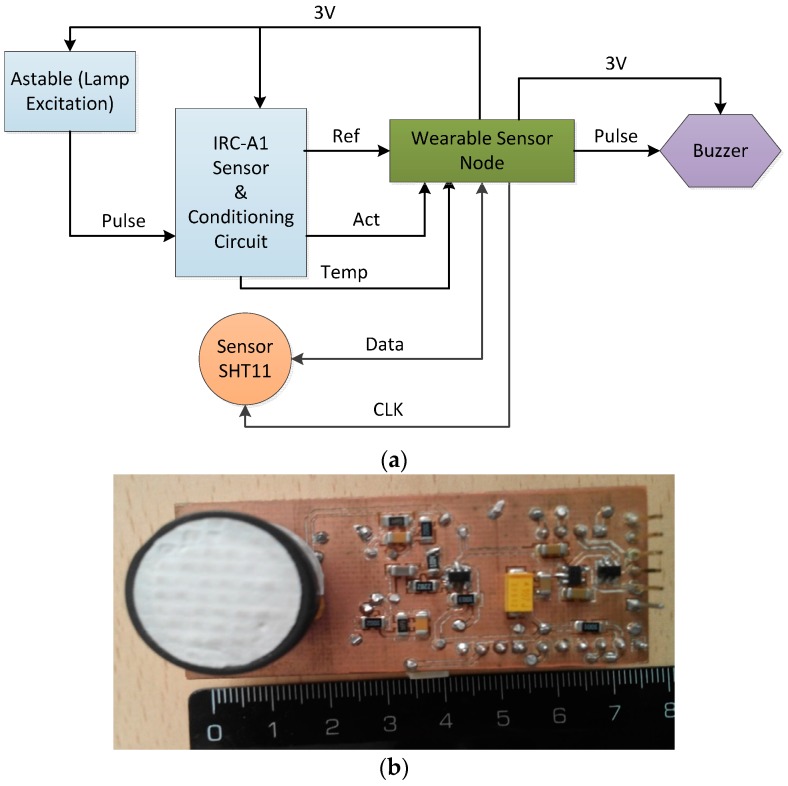
Sensor board and node connection: (**a**) block diagram; (**b**) photograph.

Sensors are housed on a specific printed circuit board (Figure 3) connected to the sensor node through a pin in-line connector. This allows an easy replacement for damaged or obsolete sensors without modifying the main node architecture. The version herein presented measures of humidity, temperature and CO_2_ concentration. A SHT11 (Sensirion AG, Zurich, Switzerland) provides the relative humidity and temperature information, with an operation range of 0% to 100% and −40 °C to 124 °C, respectively. The gas detection is performed by a non-dispersive infrared IRC-A1 CO_2_ sensor from Alphasense (Essex, UK) [12,16] in the range of 0 to 50,000 ppm to match safety applications. This sensor requires 2 Hz square wave excitation, a preheating time of 30 min and up to 40 s of response time. Our conditioning circuitry has been adapted from [17], to fit the µC analog-to-digital converter (ADC) input voltage requirements.

The 2-Hz square signal required by the sensor is directly provided by an independent astable circuit based on operational amplifier MAX4038 (Maxim Integrated, San José, CA, USA) instead of a digital output from the microcontroller. Thus, the microcontroller can be set to a low-power mode, reducing the energy consumption, while the sensor keeps always active to minimize its response time.

The three signals (reference, active and temperature voltages) in the IRC-A1 sensor must be used to calculate the CO_2_ concentration, by applying the equations given in the sensor datasheet. However, due to the complexity of the required equations and the use of a low-cost microcontroller as a sensor node manager, the microcontroller includes look-up table (LUT) data that allows for a coarse estimate of the measured CO_2_ levels, while an acoustic alarm using a buzzer included in the board is activated when concentration exceeds a predetermined level.

### 3.2. Wearable Sensor Node Software

**Figure 4:**
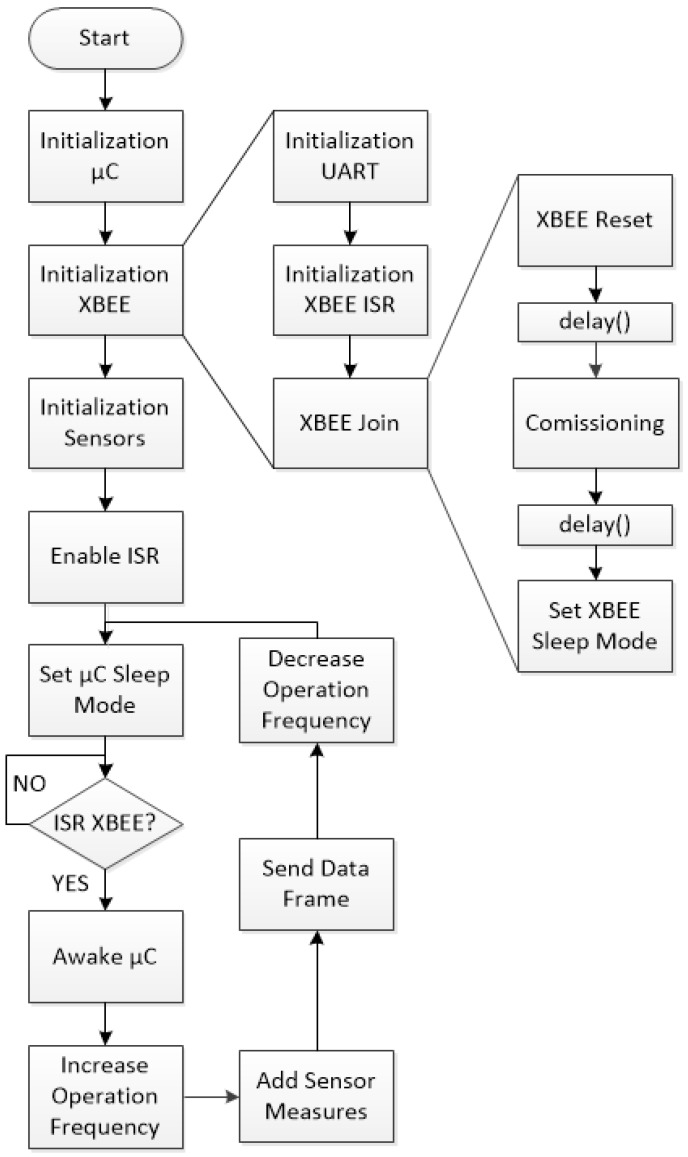
Flowchart of sensor node software operation. ISR: Interrupt service routine.

Figure 4 shows the operation flowchart of the wearable sensor node software. Once the power is switched on for the very first time, the microcontroller is firstly configured using locally-stored default parameters: duty/sleep timing, operation frequencies, etc. Next, the RF transceiver is initialized following a microcontroller request, initializing the universal asynchronous receiver-transmitter (UART), configuring the interrupt service routine (ISR) and starting the wireless network joint process. Network joining requires an XBee software reset, then a commissioning command is sent to the network coordinator node to indicate that a new device is joining the network, and finally the transceiver is sent to sleep mode. The delays shown in the flowchart are required by the transceiver for a suitable processing of the commands.

Once both microcontroller and transceiver have been initialized, sensor input/outputs and peripherals (such as the analog-to-digital converter) are configured. Configuration is completed by enabling the microcontroller to receive interrupts from the transceiver, then driving the node to sleep mode. Once fully configured, the node operation consists of a main loop, where the microcontroller is awakened by an interrupt request from the XBee. Next, the operation frequency increases in order to reduce the acquisition time, the sensor data are collected, compared to the values stored in the LUT and sent to the network coordinator through the transceiver. Finally, the operation frequency is decreased and the system goes back to sleep mode until a new XBee interrupt is produced.

## 4. Network Test

**Figure 5:**
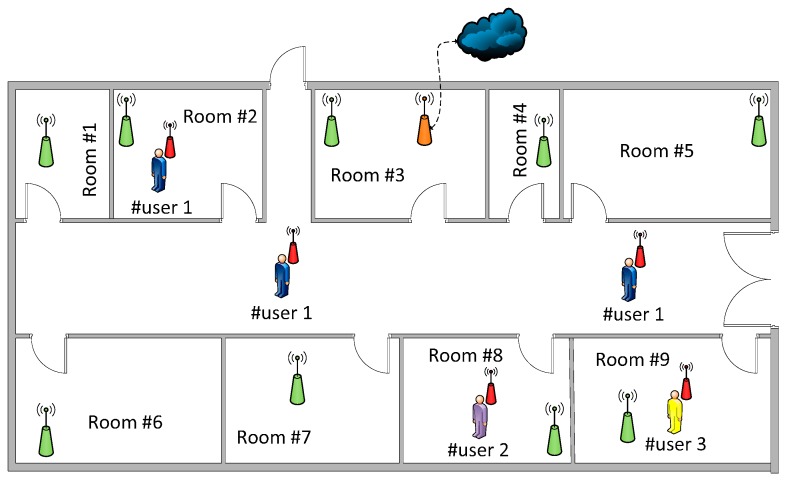
Indoor wearable wireless sensor network (W-WSN) deployment in the application test scenario (dimensions: 50 m × 15 m).

The proposed system has been tested in the indoor deployment shown in Figure 5. Each room includes a static node (green), which sends ambient parameters through multi-hop transmission to the node coordinator (orange, room #3), and then information is sent to a host device.

Wearable nodes (red, Figure 5) connect to the deployed network through the nearest fixed device, therefore providing information about the owner position and displacement. Because data transmissions are performed over short distances, RF transmission power in mobile devices is set to the minimum available value, i.e., 10 dBm for XBee modules [18]. Node locations are upgraded every time the network nodes send data to the host system. Busy periods are configured every 15 s, so the all the monitored parameters are sent from the wearable and static nodes via the host system to the processing application which processes the data, activating suitable protocols in case of unusual magnitude levels.

**Figure 6:**
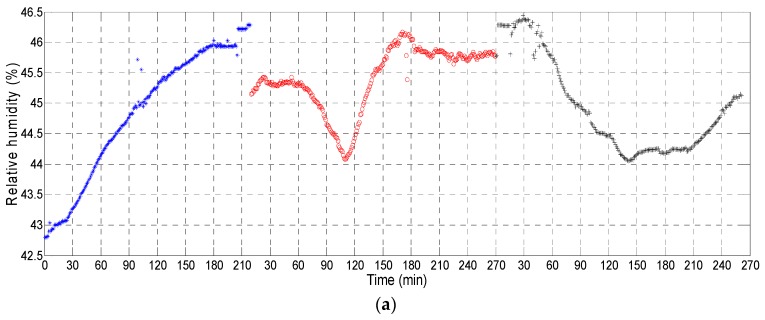

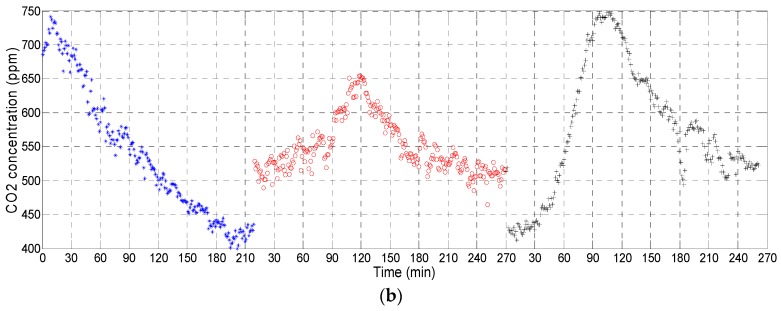
(**a**) Relative humidity acquired during laboratory sessions; (**b**) CO_2_ concentration acquired during these same periods.

To check the correct operation of the proposed W-WSN, wearable nodes were positioned on volunteers moving along the monitored area. Figure 6 shows the measurements provided by one of the volunteers wearing a node for three consecutive days, for periods of 3.5–4.5 h. Figure 6a presents measurement results for humidity, while Figure 6b shows the CO_2_ concentration. CO_2_ peaks correspond to measurements when the user entered one of the dependencies occupied by 26 students.

To validate the network link quality, the different packet error ratios (PER) have been determined. The results (Table 1) show a PER for static nodes higher than in the case of mobile nodes connected to static devices, mainly due to the fact that static links are in non-line of sight, while the mobile nodes are connected to the network with line of sight. The increase of errors in packet transmission due to node reconnections is related to changes in user location.

**Figure 7:**
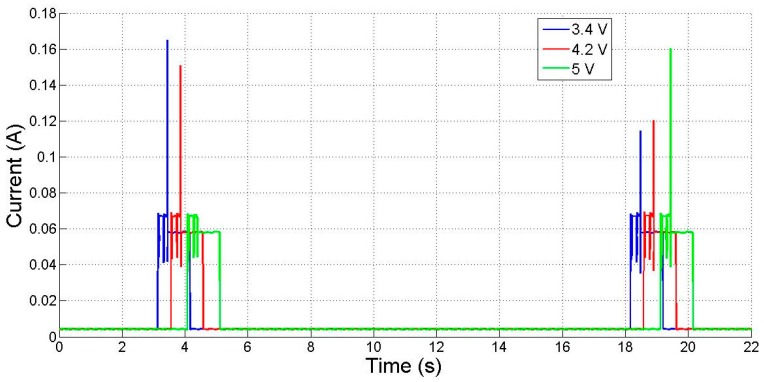
Mobile node current consumption. Nodes are biased by a nominal 3.7 V battery, charged to 5 V (**green**), 4.2 V (**red**) and 3.4 V (**blue**).

An important issue in the design of a wearable wireless sensor node is its operating lifetime. To obtain a reliable node lifetime estimation, the evolution of the battery voltage and energy level in real operation conditions has been measured and numerically modeled. First, it was verified that the experimental power consumption is constant in each one of the node states along time. Then, to obtain the battery discharge for the 3.7 V, 800 mAh LiPo LP-573442-1S-3 battery, the current node consumption profile was measured for several battery voltage values (Figure 7). As the figure shows, the current consumption is also independent of the battery output voltage: 5.5 mA in sleep mode (mainly due to keeping the CO_2_ sensor powered to allow fast measurement without need for warm-up time), 69 mA in acquisition mode and 60 mA in RF transmission. This is due to the use of LDO voltage regulators to power the node at fixed voltages from the battery varying voltage.

**Figure 8:**
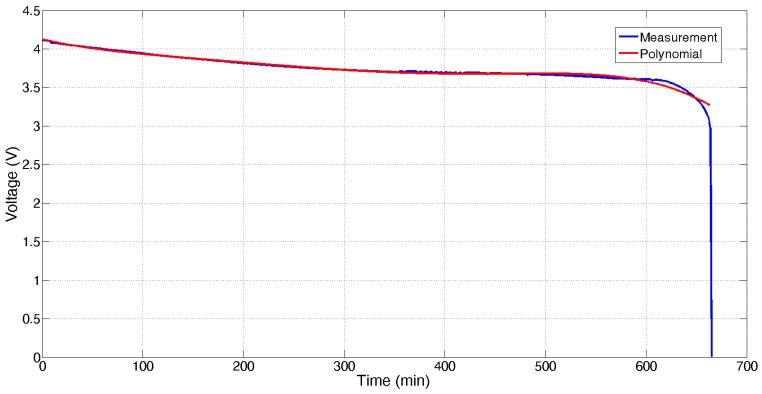
Experimental battery voltage (V) vs. time (min) for a constant 60 mA discharge.

The battery discharge at constant current is shown in Figure 8: the battery voltage drastically drops after its value falls below 3.5 V. For the useful region, it is modeled according to

(1) $$v\left(t\right)=\;-1.08\times 10^{-13}\times t^{5}+1.493\times 10^{-10}\times t^{4}-7.36\times 10^{-8}\times t^{3}\;+1.729\times 10^{-5}\times t^{2}-3.047\times 10^{-3}\times t+4.125\;\;(v\;\text{in volts},\;t\;\text{minutes})$$

**Figure 9:**
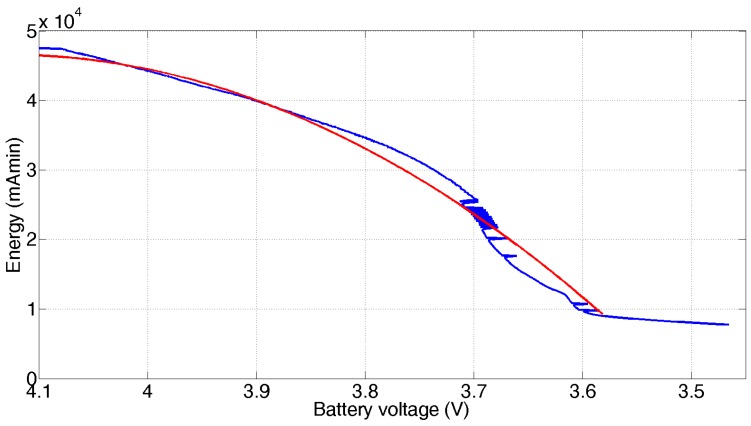
Experimental (blue) and polynomial (red) fit for battery discharge (mAmin) vs. battery voltage.

Based on the results presented in Figure 8, the relationship between the remaining energy and the voltage provided by the battery can be obtained. The result is presented in Figure 9. This behavior is approximately given by:

(2) $$C\left(v\right)=\;-1.243\times 10^{5}\times v^{2}+1.026\times \;10^{6}\times v-2.073\times \;10^{6}\;(v\;\text{in volts},\;C\;\text{in mAmin})$$

On the other hand, the equation of the node battery discharge in each working cycle can be obtained according to the process described in [15] as follows

(3) $$C=\int_{t_{0}}^{t_{1}}i_{1}(t)dt+\int_{t1}^{t_{2}}i_{2}(t)dt+K\int_{t2}^{t_{3}}i_{3}(t)dt$$

where *i_x_(t)* represents the current in the three different node states: sleep, with time limits *t*_0_ and *t*_1_; measure, with time limits *t*_1_ and *t*_2_; and RF transmission, with time limits *t*_2_ and *t*_3_. Since the current consumption in each state (see Figure 7) is constant, Equation (3) can be simplified to

(4) $$C=i_{1}\left(t_{1}-t_{0}\right)+i_{2}\left(t_{2}-t_{1}\right)+i_{3}\left(t_{3}-t_{2}\right)$$

In this way, it is possible to estimate the battery discharge as a function of time as

(5) C(t)= −6.575×t+47998.38 (C in mAmin, t in minutes)

**Figure 10:**
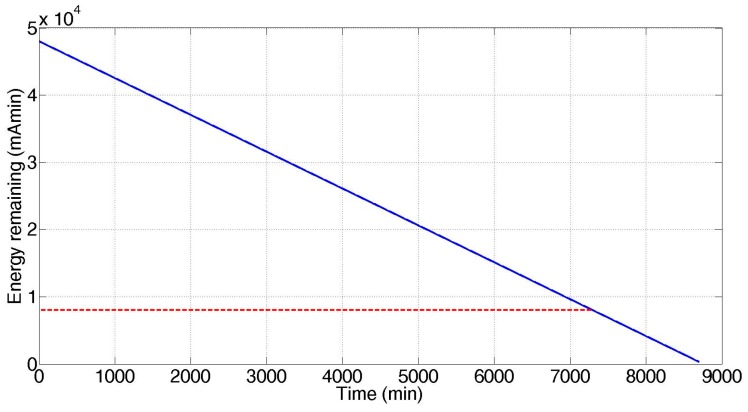
Lifetime estimation. Red line indicates the useful energy limit stored in the battery.

Taking into account that the useful operating voltage is limited to 3.5 V, which corresponds to a remaining energy of approximately 8000 mAmin (Figure 9), simulation results indicate a wearable node operating life of 5.07 days (Figure 10), close to the 5.34 days experimentally determined, for a duty cycle of 73.1 ms and 15 s of working cycle. These values ensure a suitable operating life for a wearable device, allowing setting down an appropriate schedule for battery recharge processes, i.e., this operability time is long enough for a portable system to be charged on a daily basis. The rechargeable system integrated in mobile sensor node is essential for this application and it is an improvement with respect to previous work.

Finally, Table 2 summarizes the main performances of the proposed wearable node and compares them with other similar solutions.

## 5. Web Data Collection and Processing

To attain a complete and versatile system, a remote web application has been developed. A wireless sensor network is a paradigm belonging to the widest concept of Internet of Things (IoT). Therefore, proper use of the data provided by the sensors will require the application of computational methods based on cloud computing and Big Data techniques [19,20]. Following this new paradigm, handling large amounts of information requires the use of non-relational databases. In this work, the open-source MongoDB database [21] has been selected. MongoDB is a high performance database, with high availability and automatic information scaling stored in JavaScript Object Notation (JSON [22]) format. Therefore, it is resilient to dynamic changes in the data structure. This is an important feature because as the sensors installed in the nodes could be changed at any time, the underlying data model must be capable to support such changes in a transparent manner.

**Figure 11:**
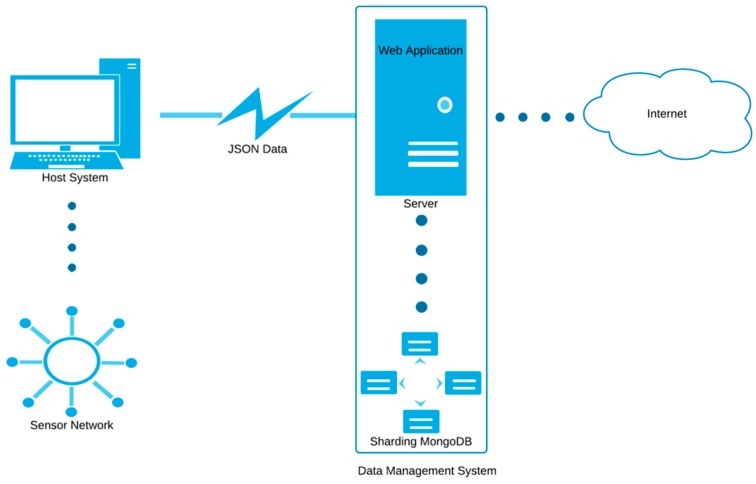
Interconnection of web application elements. JSON: JavaScript Object Notation.

The management and monitoring of the proposed sensor system is based on a distributed architecture, with two clearly defined roles: network control; and data collection, processing and display (Figure 11).

Network control is provided by a host system connected to the sensor network through an USB-to-XBee adaptor. This host is a proxy between the sensor network and the data management system. It receives data from the network (sensor measurements and node network locations), and sends commands and parameters to the full system or to specific sensors. The host system broadcasts node discovery requests to the network periodically in order to determine in real time the mobile node location.

**Figure 12:**
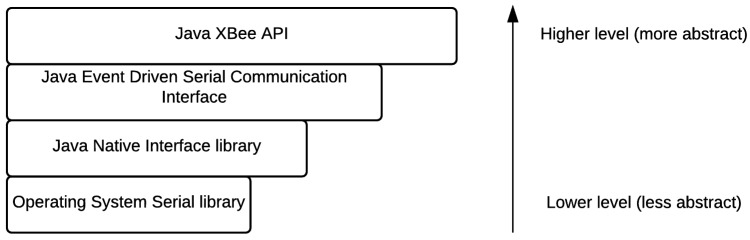
Abstraction layers from the operating system to the application code. API: Application program interface.

**Figure 13:**
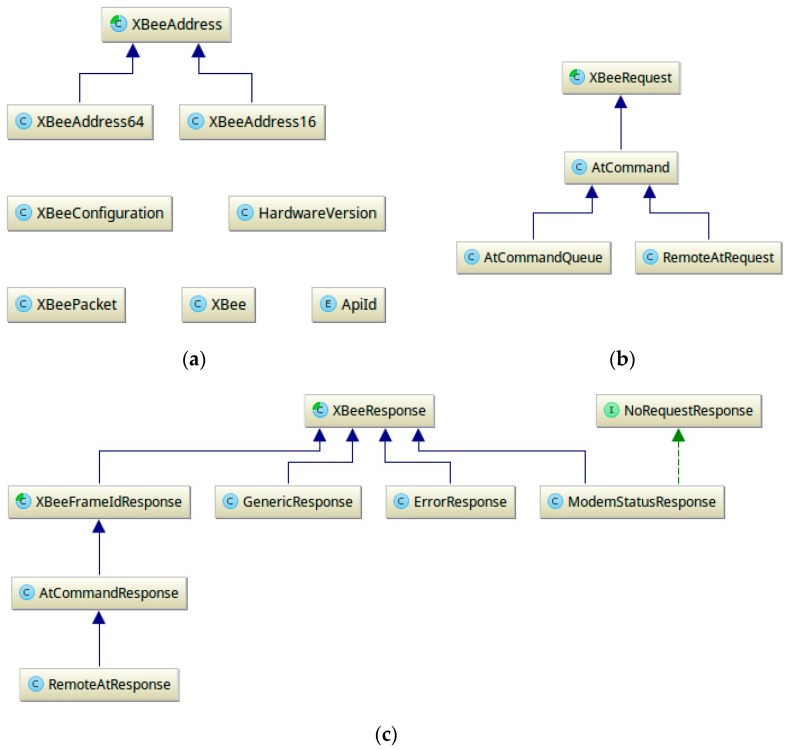
(**a**) Class hierarchy modelling the XBee according to the datasheet; (**b**) Class hierarchy modelling the XBee request according to the datasheet; (**c**) Class hierarchy modelling the XBee response according to the datasheet.

Commands to the sensor nodes use a high level library, abstracting the lower level details of the communication protocol (Figure 12) and encapsulating the DigiMesh frame model (Figure 13), therefore making it transparent to the end user.

The data management system receives data from the sensor network through the host system via hypertext transfer protocol (http), formatting data in JSON. This allows sending and receiving of structured data using plain text, which simplifies and lightens the processing. JSON has many advantages over other existing formats, such as EXtensible Markup Language (XML [23]), requiring less boilerplate text to represent the data and a human-readable structure. Data management is divided in two different parts, kept in the same physical system for simplicity: the web application and the information storage system.

**Figure 14:**
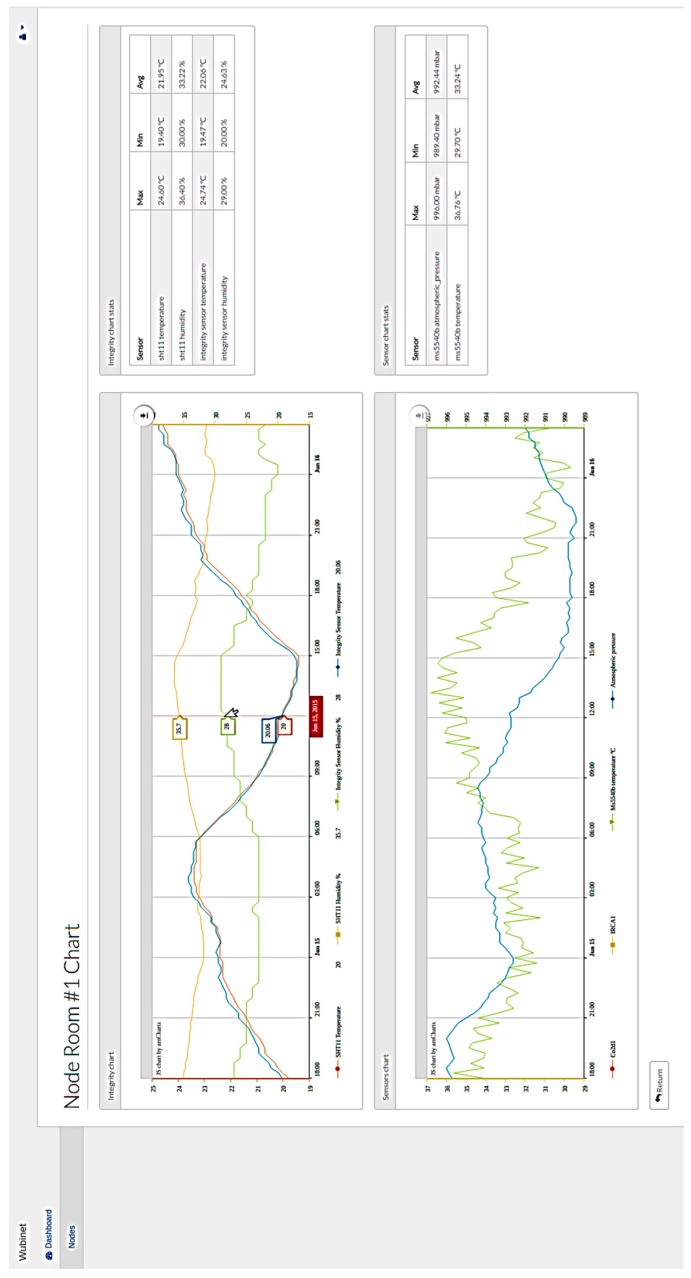
Web application screenshot.

The web application (Figure 14) allows the user to have a graphical view of the data collected by the network, enabling dynamic monitoring of the network state. It also permits the modification of network parameters such as the duty cycle or the power data transmission. This developed application is based on an event-driven model, where network control tasks are executed asynchronously according to the dynamic events generated by the network, mainly the sent and received packets. The most important feature of the view layer is the ability to plot the network data gathered from the sensors. The view layer relays on an application program interface (API) that allows sending of information using JSON format, easily exportable to other systems. On the other hand, a powerful JavaScript charting library has been used to plot the network data in a timeline. The view layer code plots the measurements by sensor node using a different scale per parameter. This representation allows the user to analyze the timeline at a glance. In addition, the software is able to refresh the charts in a timely manner with the new measures generated from the nodes.

## 6. Conclusions

This work presents an indoor smart environment monitoring system for safety applications. It is based on custom wearable sensor nodes, connected to a static WSN. Through a web application the network configuration can be controlled and managed remotely, while receiving and representing the information collected by the nodes. The system has been developed for a hazardous gas environment, but could be applied to a number of other safety applications or in other areas such as the tracking of medical devices in a hospital.

## Figures and Tables

**Table 1:** Packet Error Ratio.

Node	Rate (%)
Static Node	2.6
Mobile Node Connected to Static Router	0.95
Mobile Node Reconnecting to Other Router	3.4

**Table 2:** Sensor Nodes Comparison.

Device Parameters	IRIS Crossbow	Micaz Crossbow	TelosB Crossbow	Waspmote Libelium	This Work
**Processor**					
Microcontroller	ATMega1281	ATMega1281	MSP430	ATMega1281	PIC18F26J50
N° Bits	8 bits	8 bits	16 bits	8 bits	8 bit
Frequency	N/A	N/A	N/A	8 MHz	8 MHz
Active Mode Current	8 mA	8 mA	1.8 mA	9 mA	7 mA
Sleep Mode Current	8 µA	<15 µA	5.1 µA	62 µA	<6 uA *
**RF Transceiver**					
Frequency Band	ISM 2.4 GHz	ISM 2.4 GHz	ISM 2.4 GHz	ISM 2.4 GHz	ISM 2.4 GHz
Outdoor Range	>300 m	75–100 m	75–100 m	750–1500 m	750–1500 m
Indoor Range	>50 m	20–30 m	20–30 m	60–90 m	60–90 m
Sensitivity	−101 dBm	−94 dBm	−94 dBm	−100 dBm	−100 dBm
Max. Tx Power	3 dBm	0 dBm	0 dBm	18 dBm	18 dBm
Receive Mode	16 mA	19.7 mA	23 mA	57.08 mA	56.4 mA
Transmission Current	17 mA	17.4 mA	N/A	188 mA	69 mA
Sleep Mode	NA	1 µA	1 µA	120 µA	<12 µA
**Power Supply**					
Battery	2 × AA batteries	2 × AA batteries	2 × AA batteries	N/A	Li-Po
External Power	2.7 V to 3.3 V	2.7 V to 3.3 V	N/A	3.3 V to 4.2 V	3.5 V to 4.2 V

* Measures without sensors, but including battery charger circuit. N/A: Not Available.
